# Critical review of the impact of core stability on upper extremity athletic injury and performance

**DOI:** 10.1590/bjpt-rbf.2014.0108

**Published:** 2015-09-01

**Authors:** Sheri P. Silfies, David Ebaugh, Marisa Pontillo, Courtney M. Butowicz

**Affiliations:** 1 Department of Physical Therapy & Rehabilitation Sciences, Drexel University, Philadelphia, PA, USA; 2 Department of Health Sciences, Drexel University, Philadelphia, PA, USA; 3 Penn Sports Medicine, Good Shepherd Penn Partners, Philadelphia, PA, USA

**Keywords:** core stability, neuromuscular control, athletic injuries, athletic performance

## Abstract

**BACKGROUND:**

Programs designed to prevent or rehabilitate athletic injuries or improve athletic performance frequently focus on core stability. This approach is based upon the theory that poor core stability increases the risk of poor performance and/or injury. Despite the widespread use of core stability training amongst athletes, the question of whether or not sufficient evidence exists to support this practice remains to be answered.

**OBJECTIVES:**

1) Open a dialogue on the definition and components of core stability. 2) Provide an overview of current science linking core stability to musculoskeletal injuries of the upper extremity. 3) Provide an overview of evidence for the association between core stability and athletic performance.

**DISCUSSION:**

Core stability is the ability to control the position and movement of the trunk for optimal production, transfer, and control of forces to and from the upper and lower extremities during functional activities. Muscle capacity and neuromuscular control are critical components of core stability. A limited body of evidence provides some support for a link between core stability and upper extremity injuries amongst athletes who participate in baseball, football, or swimming. Likewise, few studies exist to support a relationship between core stability and athletic performance.

**CONCLUSIONS:**

A limited body of evidence exists to support the use of core stability training in injury prevention or performance enhancement programs for athletes. Clearly more research is needed to inform decision making when it comes to inclusion or emphasis of core training when designing injury prevention and rehabilitation programs for athletes.

## Introduction

Prevention and treatment of musculoskeletal injuries in athletes is a principal concern of coaches, medical and fitness professionals, and athletes themselves. It is estimated that greater than 10,000 individuals a day seek medical attention for sports, recreation, and/or exercise related injuries^1^. The National Collegiate Athletic Association Injury Surveillance System sites over 11,000 injuries per year^2^. A study utilizing injury data from the International Association of Athletics Federation of World Athletic Championships suggests acute non-contact injuries comprise 13% and overuse injuries 44% of competition injuries^3^. Junge et al.^4^ reported that 22% of all documented injuries in the 2008 Summer Olympic Games were non-contact, overuse injuries. At the collegiate level, an estimated one third of all athletic injuries are non-contact in nature, with 20% on average involving the upper extremity^2^. These data have prompted National and International associations to recommend future studies to investigate circumstances and characteristics of non-contact injuries in greater detail with a goal of identifying possible risk factors and focusing initiatives toward injury prevention^2^.

Over a decade ago, core stability was proposed to play an important role in athletic injury and performance^5^^,^ 6. It was hypothesized that poor core stability increased the risk of upper extremity athletic injuries^5^ and negatively affected athletic performance^5^^,^ 7 ^,^ 8. This hypothesis was readily accepted despite the lack of consensus definition of "core stability" or a robust body of scientific evidence to support it. Today the acceptance of training the "core" as part of injury prevention or rehabilitation programs for athletes is pervasive. However, is there sufficient evidence to support this practice?

The objectives of this paper are to: 1) open a dialogue on the definition and components of core stability, 2) provide an overview of current scientific evidence linking core stability to musculoskeletal injuries of the upper extremity, and 3) provide an overview of evidence for the association between core stability and athletic performance. Additionally, we will identify clinical tests and measures that might assist in capturing core stability status and discuss gaps in the evidence for the purpose of informing future research.

## Definition of the core and core stability

A number of definitions have been proposed to describe the "core". A commonly accepted definition is the boney skeleton, ligaments, and musculature of the lower spine, pelvis, hips, and proximal lower extremities^5^^,^ 9. When considering overhead athletes, this definition has been expanded to include the boney skeleton, ligaments, and musculature of the shoulder girdle^5^. Therefore, from this perspective, key core musculature includes muscles in the superficial and deep abdominal wall, pelvic floor, erector spinae, and segmental back muscles, as well as those supporting the pelvic girdle/hip and scapula.

"Core stability" has been defined as the ability to control trunk position and motion for the purpose of optimal production, transfer, and control of forces to and from the terminal segments during functional activities^5^. The concept of stability encompasses both static and dynamic control. This includes the ability of the neuromuscular system to keep the trunk in (or return it to) an upright position (static) and control trunk movements (dynamic). This is predominantly accomplished via quick postural responses by the neuromuscular system to both internal and external perturbations (expected or unexpected). This also includes perturbations caused by forces generated from or traveling through the extremities. Both feed-forward and feed-back mechanisms are integrated within the neuromuscular system to respond to these forces^10^.

**Figure 1.**
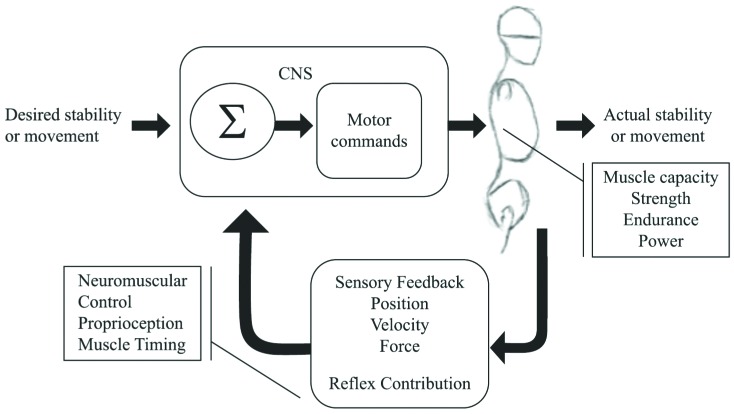
A schematic of the components of a well-functioning neuromuscular system. The boxes representing muscle capacity and neuromuscular control include examples of parameters that can be measured to assess these components. The central nervous system (CNS) includes the integration (S) of the sensory information and determination of the motor command.

A well-performing neuromuscular system is essential for core stability. This system provides stability by relaying available sensory information (position, velocity, force) to the central nervous system (CNS), which then activates appropriate musculature to generate forces quickly and accurately^11^ (Figure 1). This indicates that core stability is a dynamic process that requires optimal muscle capacity (strength, endurance, power) and neuromuscular control (accurate joint and muscle receptors and neural pathways) that can quickly integrate sensory information and alter motor responses relative to internal and external information.

## Scientific evidence

Two literature searches from January 1990 to December 2014 were conducted within the CINHAL, MEDLINE, and SPORTDiscus databases. These searches were supplemented by reviewing the reference list of articles that met our criteria. The first search was intended to identify prospective and longitudinal studies that assessed the relationship between core [search terms: core, trunk, lumbopelvic spine] stability [stability, strength, neuromuscular control] and shoulder, elbow, or wrist injury [injury, pain] in athletes [athlete]. We included case control or cohort studies (injured vs. non-injured athletes). However, we did so with the understanding that we could not separate coincidental relationships from causal ones. Systematic reviews were used when available and weighted more heavily in our summary of the evidence. Based upon our operational definition of core stability, we included articles that assessed both sensory and motor aspects of neuromuscular control as well as core muscle strength and endurance. Articles that included interventions had to clearly incorporate specific exercises for and measure changes in core stability within a randomized clinical trial (RCT). The findings of intervention RCTs that focused on prevention of injury and where the injury group was limited to those with non-contact injuries were more heavily weighted in our conclusions. Our literature search identified 64 potential articles; however, this reduced to seven when we applied our criteria for region of injury, subject population, study design, and measured variables of core stability.

The second search was intended to identify articles that assessed the relationship between core stability [same search terms as above] and athletic performance [performance]. We included intervention studies on healthy athletes in which muscle capacity or neuromuscular control training was instituted to determine if changes in performance were associated with changes in specific core stability measures. High quality RCTs and systematic reviews were weighted more heavily in our conclusions. This search resulted in 109 articles that were narrowed to 11 based upon our criteria.

## Evidence linking core stability to upper extremity athletic injuries

**Figure 2.**
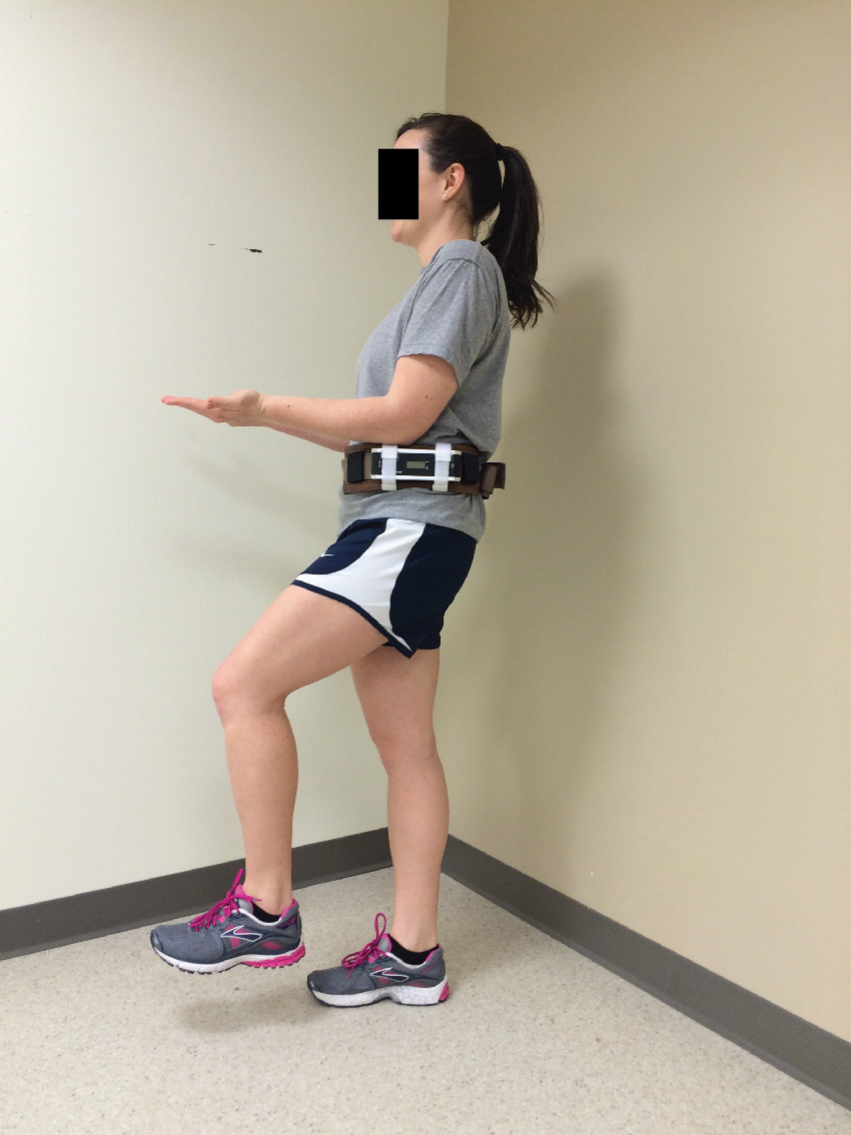
Example of the single leg raise test to assess the ability to control the lumbopelvic region when moving into unilateral stance as described by Chaudhari et al.^14^.

**Figure 3.**
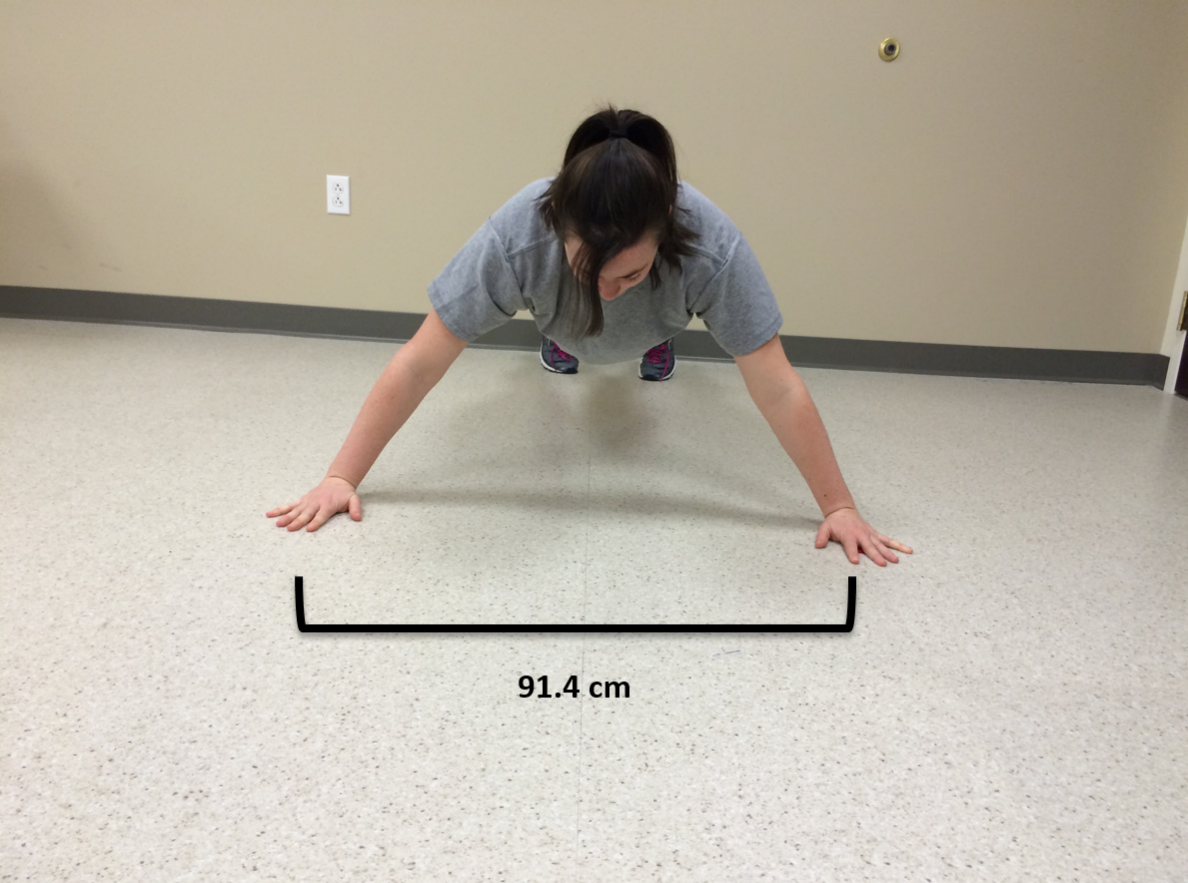
Start position for the performance on the Closed Kinetic Chain Upper Extremity Stability Test as described by Pontillo et al.^17^.

Deficits in core stability have been proposed to lead to shoulder^5^^,^ 12 or elbow injuries^13^. Although our search failed to identify any systematic reviews on this proposed relationship, it did identify three recent prospective injury risk studies. Chaudhari et al.^14^ investigated the association between lumbopelvic control and injuries in baseball players. Lumbopelvic control was assessed during a single-leg raise test in 347 professional baseball pitchers during spring training. This test was performed in standing with the athlete attempting to keep the waist as level as possible while they slowly lifted one foot up as though they are going to step up onto a curb^15^(Figure 2). Days missed because of injury during the season were tracked for each player. They found that pitchers with less control during the single-leg raise task (highest tertile of anterior-posterior lumbopelvic motion) were 3 times more likely to miss at least 30 days than those pitchers demonstrating lower amounts of lumbopelvic motion. Non-contact injuries of the upper extremity and trunk/back accounted for 60% and 14% of the injuries reported over the season, respectively. However, Endo and Sakamoto^16^ reported no relationship between core muscle endurance (prone bridge, side bridge) and shoulder or elbow injury in junior high school baseball players. Pontillo et al.^17^ reported on a prospective injury risk study of Division 1 American football players. Their data indicated that players who suffered a shoulder injury during the season could be identified by preseason performance on the Closed Kinetic Chain Upper Extremity Stability Test (CKCUEST). The CKCUEST is performed in a prone plank position where the athlete is asked to alternate periods of upper limb single support to touch one of two lines placed 91.4 cm apart over a 15-second time period^18^ (Figure 3). Based on their findings, they determined that a cut score of less than 21 touches could identify athletes at risk for future injury. Collectively, these studies suggest that poor core stability (as measured by the CKCUEST and single-leg raise tests) should be considered a potential risk factor for future upper extremity injury.

**Figure 4.**
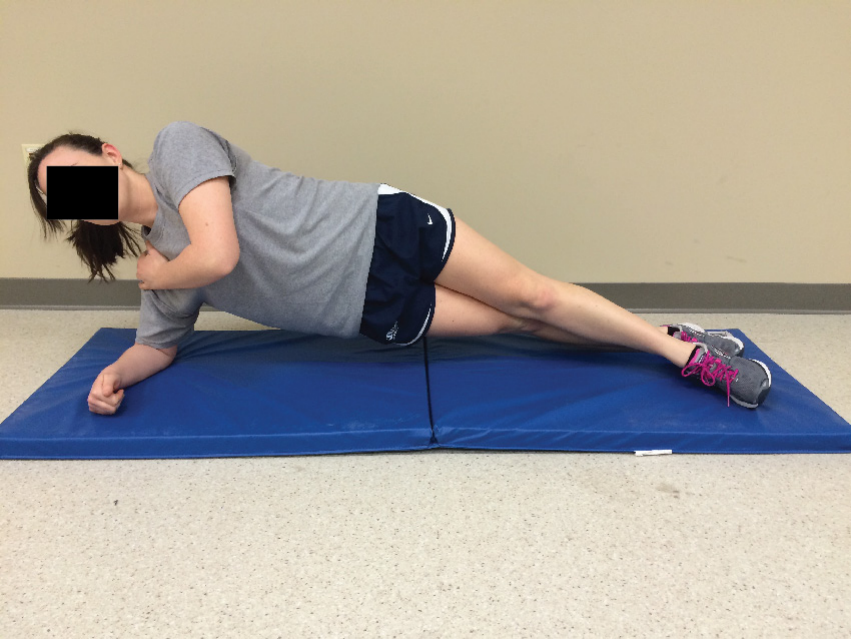
The side-bridge endurance test position as described by Tate et al.^19^.

Two case control and cohort studies of swimmers also lend support to the hypothesized relationship between impaired core stability and upper extremity injuries. Tate et al.^19^assessed scapular dyskinesia, core muscle endurance (side-bridge, prone bridge), and the CKCUEST on 236 female youth, high school, or US Masters swimmers. They compared test findings between subjects who reported substantial shoulder disability and pain (Penn Shoulder Score and the sport performance module of the Disability of the Arm, Shoulder and Hand Outcome Measure) and those who did not. Neither the observation of scapular dyskinesia nor reduced core endurance was more predominate in the group with shoulder pain or disability, with the exception of reduced side-bridge endurance^20^(Figure 4) for high school age swimmers. Harrington et al.^21^ also assessed core endurance in swimmers with and without shoulder pain (NCAA Division 1 females, n=37). They reported no significant differences in core endurance for side-bridge and prone bridge between the groups. Together these two studies offer conflicting support for poor core stability being associated with shoulder pain in competitive swimmers.

**Figure 5.**
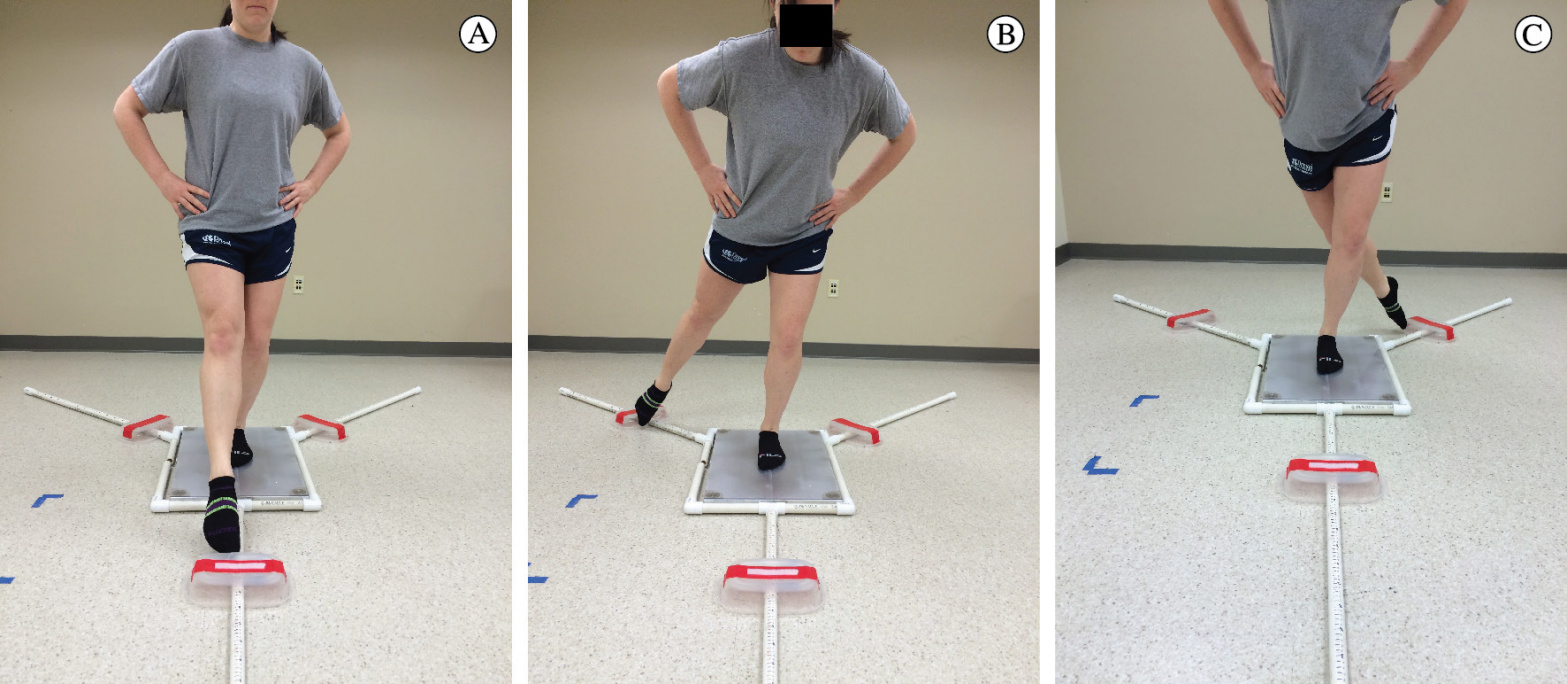
The Y-balance test performed in the (A) anterior, (B) posterior medial, and (C) posterior lateral directions.

Based upon the kinetic chain theory^5^, abnormal neuromuscular control in any portion of the chain could alter forces and biomechanics during upper extremity movements; therefore we included these two recent cohort studies that also assessed standing balance. Radwan et al.^22^ tested 61 Division III overhead athletes, 14 with current shoulder pain and dysfunction from a non-contact injury. Core stability tests included double leg lowering test (DLLT), Sorensen modified extensor endurance test, and side plank and the single leg balance test (SLBT). Only the SLBT stance time, which assesses static balance, was significantly reduced in the shoulder pain group. Garrison et al.^23^ also reported significantly decreased dynamic balance in both lead and stance legs, as measured by the Y-balance test^24^ (Figure 5), in high school and collegiate baseball players with ulnar collateral ligament (UCL) tears. Their study included 30 male athletes with an UCL tear and 30 non-injured, age-, arm dominance-, experience-, and position-matched controls. Although these studies do not directly support decreased standing balance control as a cause for shoulder injury/pain or UCL tears, they do support the hypothesis of a potential relationship between poor core control, as measured by clinical tests (SLBT, Y-balance), and upper extremity injury.

## Is the evidence for an association between poor core stability and upper extremity injury different than that found for the spine and lower extremity injuries?

While not the focus of the review, it seems important to summarize briefly the evidence for an association between core stability status and spine and lower extremity (LE) non-contact injuries. Based on data from Hootman et al.^2^, the percentage of injuries to the spine and LE during practice and games averages 11.5 and 53%, respectively. Thus, it is not surprising that a majority of research related to core stability and injuries has focused on the LE. Several prospective, longitudinal studies from a large cohort of Division 1 collegiate athletes (n= 277) support the relationship between core stability and LE injury^25^^,^ 26. These studies indicate that decreased trunk proprioception and neuromuscular control are predictive of future knee injuries in female collegiate athletes. Using this same cohort of athletes, Cholewicki et al.^27^found decreased trunk neuromuscular control (delayed trunk reflexes) to be predictive of future low back injury (LBI). However, neither decreased trunk proprioception nor trunk muscle activation imbalance were predictive of LBI in this cohort^28^^,^ 29. In another large (n=162) prospective study, Nadler et al.^30^found an association between hip muscle strength imbalance and LBI in female athletes. A number of studies have suggested that impaired hip muscle strength is associated with LE, particularly knee injuries^31^^,^ 32. Dynamic balance impairment as measured by the Star Excursion Balance Test, Single-leg Hop for Distance, and Lower Extremity Functional Test have also been associated with back, knee, and ankle injuries^24^^,^ 33 ^,^ 34. Work in this area has also advanced to include studies that suggest core stability training can reduce the risk of LE injuries^35^^-^ 37.

While there are studies with stronger and more consistent evidence that support a relationship between core stability and LE or back injuries, these studies are not without limitations. In general, the findings associated with LE injury are weighted to studies that only measured muscle capacity and stronger findings are associated with knee injuries in female athletes. The primary limitation of these findings is that it is unclear how much poor core stability contributes to injury risk in light of other risk factors.

## Evidence linking core stability to athletic performance

Athletic performance can be assessed through functional, agility, speed, accuracy, and power tests that involve the upper extremity, LE, or the entire body. Based on the kinetic chain theory, a "break in the chain"^38^should lead to a decrease in optimal force generation or efficiency, and subsequent decrease in performance. Our search on this topic revealed one systematic review by Reed et al.^39^. Their inclusion criteria were targeted toward core training (isolated or integrated into a rehabilitation program for injured athletes), measurement of sports performance, and subjects under the age of 65 years old. Their search produced 10 RCTs and 14 non-randomized trials. An example of a study included in this review was an eight-week core endurance training protocol completed by Tse et al.^8^. This non-randomized intervention study investigated the effect of training on various measures of athletic performance (vertical and broad jump, shuttle run, 40-meter sprint, overhead medicine ball throw, and ergometer test). While the authors report significant improvement in the side-bridge endurance, they found no difference between the control group and the group performing core endurance training on any of the performance measures. However, this may be attributed to the fact that only core muscle endurance was trained and not aspects of neuromuscular control.

The diversity of definitions and assessments of core stability, as well as the diversity in core training regimens, hampered summarizing findings of this systematic review. The largest group of studies assessed the effect of core strengthening on LE performance. These studies demonstrated mixed results with 3 of 10 studies reporting increased running performance post core training and 2 reporting no change. However, in general those studies reporting change were conducted using active adults and not trained athletes^39^. The 6 studies that evaluated aspects of upper extremity performance, suggest that core muscle endurance is not a strong predictor of sports performance. However, a more recent RCT assessing the effects of strength training on nationally ranked junior tennis players (including core muscle exercises) found improvement in service velocity following a 6-week intervention^40^. Reed et al.^39^ suggest that isolated training of the core should not be the primary emphasis for programs with the goal of enhancing sports performance. Instead, they propose training tailored to the athlete's sport (sport-specific training), as studies using these approaches demonstrated at least improved performance in sport-specific tasks (e.g. golf club head speed, bat speed).

Reed et al.^39^ excluded studies that did not involve core training intervention. There are several non-intervention studies that have investigated the relationship between core stability and general^41^^-^ 43 and specific athletic performance^42^. Nesser et al.^41^ investigated the relationship between core stability and performance in Division I football players by measuring: 1) strength, tested by three power lifting exercises; 2) core muscle endurance; and 3) sports performance, tested via sprints of various lengths, countermovement vertical jump, and a shuttle run. Total core strength was defined as the total isometric hold times of the trunk flexion, trunk extension, and left and right side-bridge tests. The authors theorized that increases in core strength would correlate with increased strength and performance measures. Significant correlations were found between total core strength and sprints, agility tasks, 1 repetition maximum squat, and bench press. Okada et al.^42^ also compared core stability, Functional Movement Screen (FMS), and performance testing in a group of athletic subjects. However, core stability was tested with four endurance tests: sustained flexion, extension, left and right side-bridge. This study was the first to include, amongst the performance tests, an upper extremity performance test consisting of a backwards overhead medicine ball throw. The authors reported weak to moderate significant correlations between core stability measures and performance. There were no significant correlations between core stability and FMS. It should be noted that, in both studies^41^^,^ 42, the authors only measured core muscle endurance, not neuromuscular control, and the terms "stability" and "strength" were used interchangeably. Sharrock et al.^43^ used the double leg lowering test (DLLT) to assess muscle capacity of the rectus abdominis and oblique muscles and correlated this with four performance tests: forty-yard dash, T-test, vertical jump, and a medicine ball throw. The medicine ball throw was the only measure that significantly correlated with the DLTT, with an improved score on the double leg lowering correlating with an improved score on the medicine ball throw. Chaudhari et al.^44^ was the only study we found that assessed the association between neuromuscular control and in season sports performance. They assessed the anterior-posterior motion of the pelvis during the single-leg raise test in 75 Minor League pitchers and tracked their performance (innings pitched, walks + hits/ inning, strike out/inning). The group of pitchers with better lumbopelvic control demonstrated better accuracy (walks + hits/inning) and endurance (innings pitched) with trends toward difference in other measures of game performance.

Collectively, the findings from these studies imply that select measures of core stability are related to athletic performance and function. However, given the study designs, a true causal relationship cannot be strongly inferred and further research is warranted to expand upon this premise.

## Limitations and gaps in the literature

Our literature search and subsequent study selection criteria resulted in a small number of higher quality studies that addressed the relationship between core stability and upper extremity injury. Of the few prospective longitudinal studies reviewed, there is evidence to support the proposed relationship. However, sample sizes in these studies were small. More importantly, the amount of risk potentially posed by poor core stability versus other factors such as history of injury, level of recovery, specific shoulder or elbow joint ligament and muscle impairments, reduced joint motion, exposure to the sports activity (pitches throw, swimming strokes), non-orthopaedic conditions, or environmental conditions (altitude, extreme heat, or cold) has not been directly compared. Examples of the amount of risk poor core stability might possess are provided by Cholewicki et al.^27^, who reported a larger risk for future low back injury (LBI) being associated with the history of a LBI (Odds Ratio [OR] 2.84) than risk associated with decreased trunk neuromuscular control (OR 1.02). Zazulak et al.^25^ also reported that a history of LBI was the only significant factor associated with LE injuries in male athletes. Prior injuries predicting future injury generated the hypothesis that prior injury somewhere in the kinetic chain is associated with future injuries.

The study design of the majority of core stability cohort or intervention studies linked to an athletic injury does not allow us to determine if core stability deficiencies were present prior to injury, a consequence of decondition from the injury, or if the outcome of rehabilitation can be directly associated with changes in core stability alone. Based on the literature reviewed, there is little direct evidence for poor core stability as a cause of or the predominant risk factor for athletic injuries. In addition, the role of pain in muscle inhibition, altered proprioception, disrupted neural processing speed, or cognitive processing cannot be eliminated from studies that assessed the association of core stability to athletes who were currently recovering from an injury^45^^-^ 47. Therefore caution is recommended in interpreting findings from these studies.

Many of the articles assessing the effect of core stability on sports performance do not focus solely on a competitive athletic population. In particular, many studies used recreationally active students and adults to assess the role of core stability in injury or performance, which may represent greater effect size than on trained athletes. Therefore, it is difficult to directly translate the findings to competitive or highly trained athletes^39^.

Our review was also limited to studies that addressed at least one aspect of core stability as we operationally defined it. Therefore, studies that solely assessed joint ranges of motion, pain, or self-reported measures of disability as risk factors for athletic injury were not included as part of the review.

## Conclusions

Core stability is a component of many, if not all, athletic conditioning, prevention, or rehabilitation programs, despite the lack of strong evidence of a direct contribution to injury prevention or enhanced performance. Where a contribution has been demonstrated, the amount of risk potentially posed by poor core stability has not been systematically evaluated in conjunction with other identified injury risk factors. Although not specifically reviewed as part of this paper, there is strong evidence for the role of history of prior injury^25^^,^ 27 and level of recovery from prior injury as risk factors for future injury^48^^-^ 50. Other factors associated with upper extremity injury, specific to the joint or region (e.g. muscle impairment, limited or asymmetric joint motion) and those factors associated with environment or exposure (number of pitches, swimming stroke type) related to the sports activity itself, have not been investigated in conjunction with core stability. We included descriptions of clinical tests that have demonstrated potential value in predicting upper extremity injuries and provided references for their reliability. These tests serve as a starting point for clinicians and researchers focused on the treatment of upper extremity athletic injuries. Future studies should be prospective, strive for larger sample sizes, and consider assessing the relationship between risk factors for known specific injuries and exposure to determine which factors are most relevant to achieving the primary goal of reducing the number and severity of athletic injuries. This information would allow coaches, medical and fitness professionals, and athletes themselves to focus on programs designed for prevention and rehabilitation of athletic injuries.
